# Cross-Protective Capacity of Japanese Encephalitis (JE) Vaccines Against Circulating Heterologous JE Virus Genotypes

**DOI:** 10.1093/cid/cis883

**Published:** 2012-10-16

**Authors:** Elina O. Erra, Helena Hervius Askling, Sutee Yoksan, Lars Rombo, Jukka Riutta, Sirkka Vene, Lars Lindquist, Olli Vapalahti, Anu Kantele

**Affiliations:** 1 Haartman Institute, Faculty of Medicine, University of Helsinki; 2 Division of Infectious Diseases, Department of Medicine, Helsinki University Central Hospital, Helsinki, Finland; 3 Karolinska Institutet, Department of Medicine/Solna, Unit for Infectious Diseases, Stockholm, Sweden; 4 Institute of Molecular Biosciences, Mahidol University, Nakhon Pathom, Thailand; 5 Centre for Clinical Research, Sörmland County Council, Eskilstuna, Sweden; 6 Travel Clinic,Aava Medical Centre, Postitalo, Helsinki, Finland; 7 Swedish Institute for Communicable Disease Control, Solna; 8 Karolinska Institutet, Department of Medicine/Huddinge, Unit for Infectious Diseases, Stockholm, Sweden; 9 HUSLAB, Division of Virology and Immunology, Helsinki University Central Hospital; 10 Department of Veterinary Biosciences; 11 Department of Medicine, University of Helsinki, Finland

**Keywords:** Japanese encephalitis, vaccine, traveler, crossreactive immunity, flavivirus

## Abstract

Current Japanese encephalitis vaccines are derived from strains of genotype III, yet heterologous genotypes are emerging in endemic areas. Inactivated vaccines given to European travelers were found to elicit protective levels of neutralizing antibodies against heterologous strains of genotypes I–IV.

Japanese encephalitis (JE), a mosquito-borne flaviviral disease, is the leading cause of epidemic encephalitis worldwide, accounting for approximately 70 000 annual cases of clinical disease [1]. Because of the severity of this disease and lack of antivirals, vaccinations are recommended, not only for inhabitants of endemic areas but also for travelers at risk [2]. However, it may not be widely recognized that there are several genotypes of Japanese encephalitis virus (JEV) circulating in endemic areas, and their epidemiology is evolving.

Japanese encephalitis viruses are divided into 5 genotypes (GI–GV). GI and GIII have mainly been isolated in temperate and epidemic areas, whereas GII and GIV have mostly been found in tropical, endemic regions [3]; GV has only been isolated 3 times [4]. There have been several reports on GI replacing GIII as the dominant genotype in numerous regions [3, 5, 6].

All JE vaccines currently available are based on viral strains belonging to a single genotype, GIII, even if this no longer constitutes the dominant JEV type in many areas. At present, there are hardly any human data on the efficacy of the current inactivated travelers’ vaccines against the circulating JEV genotypes other than GIII. The need to assess the cross-reactive potential of the GIII-derived vaccines has become widely recognized [5–7].

We looked into the vaccine-induced cross-protection against JEV genotypes I–IV after immunization with the 2 inactivated JE vaccines (Ixiaro or Japanese Encephalitis Vaccine GCC) currently given to travelers.

## METHODS

### Study Population

The study population, previously JEV-naive adult volunteers, received a primary series with either a SA14-14-2–based (Ixiaro, Intercell AG, Vienna, Austria; n = 29) or Nakayama-based (Japanese Encephalitis Vaccine GCC, Green Cross Corp, South Korea; n = 12) inactivated GIII JE vaccine before traveling to a JE-endemic area in Asia. The same volunteers had been evaluated for their immune response against the 2 GIII vaccine strains in our previous study exploring the ability of a heterologous vaccine to boost JE immunity [8]. These vaccinees were now evaluated for the presence of neutralizing antibodies against 5 JEV strains representing genotypes I–IV. The research protocol was approved by the ethics committees supervising the investigational sites. The study (EudraCT: 2010-023300-27; ClinicalTrials.gov: NCT01386827) was registered in the databases required and performed in accordance with the principles outlined in the Declaration of Helsinki. All volunteers provided informed consent.

Eligibility criteria for the study population have been described in detail previously [8]. Moreover, to avoid cross-reactions due to preexisting antibodies, participants found to be seropositive for 1 or more of the JEV test strains before vaccination (n = 5) were excluded from the final analyses.

### Determination of the Neutralizing Antibody Responses

Serum samples were collected before vaccination (day 0) and 4–8 weeks after the last vaccine dose. The serological analyses were carried out in a blinded manner. All serum samples were tested by the plaque-reduction neutralization test (PRNT) as previously described [8] using 5 JEV target strains representing genotypes I–IV: SM-1 (GI; isolated in Thailand in 2002), B 1034/8 (GII; isolated in Thailand in 1983), Nakayama (GIII; strain in Japanese Encephalitis Vaccine GCC), SA14-14-2 (GIII; strain in Ixiaro), and 9092 (GIV; isolated in Indonesia in 1981). A PRNT_50_ titer (the reciprocal of the serum dilution that reduced the virus plaque count by 50% as compared with the virus-only controls) of ≥10 was considered protective [9].

### Statistical Analysis

Statistical analysis was performed with the R 2.13.0 software (R Development Core Team 2011). The statistical significance of differences in seroconversion rates (SCRs) was assessed by 2-sided χ^2^ tests, and in levels of neutralizing antibodies by 2-sided Wilcoxon exact tests. *P* < .05 was considered significant.

## RESULTS

### Study Group Characteristics

The background characteristics of the subjects have been described in detail previously [8]. The final study population comprised 22 female and 19 male travelers between the ages of 18 and 61 years (median age, 26.0 years). Most subjects were healthy and of Finnish or Swedish origin; 1 volunteer had asthma.

### Serological Analyses

**Figure 1.**
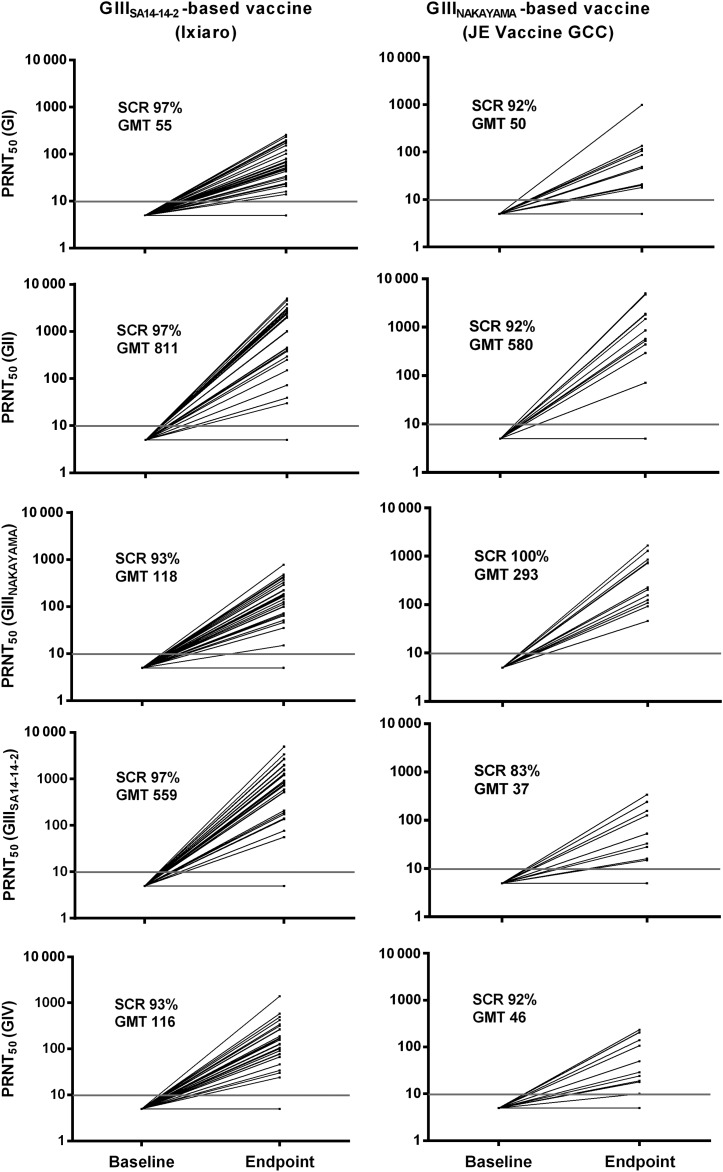
Japanese encephalitis (JE) vaccine–induced immune response in previously Japanese encephalitis virus (JEV)–naive adult travelers: PRNT_50_ titers against viral strains of different JEV genotypes are shown before and 4–8 weeks after a vaccination series with SA14-14-2–derived (Ixiaro; n = 29) or Nakayama-derived (Japanese Encephalitis Vaccine GCC; n = 12) vaccine. The gray lines indicate PRNT_50_ titer = 10. PRNT_50_ titers of ≥10 were considered protective. (PRNT_50_ titer is the reciprocal of the serum dilution that reduced the virus plaque count by 50% as compared with the virus-only controls). The seroconversion rates (SCRs) and geometric mean titers (GMTs) are given in each panel.

Figure 1 shows the individual PRNT_50_ titers of neutralizing antibodies against all target strains before and after a primary series with either of the 2 JE vaccines, and the SCRs and geometric mean titers (GMTs) attained for both vaccine groups against all 5 test strains.

Of the 29 travelers immunized with the SA14-14-2–based JE vaccine (Ixiaro), 93%–97% attained protective levels of neutralizing antibodies against the 5 JEV test strains representing genotypes I–IV. One of the subjects did not reach protective PRNT_50_ titers against any of the strains tested, and another had neutralizing antibodies to GI, GII, and homologous GIII strains (SA14-14-2) but not to heterologous GIII (Nakayama) or the GIV test strain.

Among the 12 travelers who were immunized with the Nakayama-based vaccine (JE Vaccine GCC), the SCRs varied between 83% and 100%, depending on the test strain used. All subjects exhibited a response to the GIII strain homologous to the vaccine strain (Nakayama); 1 subject failed to respond to all other strains, and 1 showed a response to all test strains except the GIII (SA14-14-2).

No significant differences were found in the SCRs between the 2 vaccine groups.

In both groups, the highest PRNT_50_ titers were presented against the GII target strain. These proved significantly higher than those against the GI, the heterologous GIII, and the GIV strains. The second highest titers were found against the test strain homologous to each vaccine strain; no difference was seen between the homologous strains and GII.

## DISCUSSION

All JE vaccines currently available are based on JEV strains isolated >50 years ago, representing only a single genotype (GIII), which no longer constitutes the dominant JEV type in many areas [3, 5, 6]. The notable changes occurring in the dynamics of the genotype distribution call for studies on the cross-reactive capacity of the current vaccines against the various genotypes.

Evaluation of neutralizing antibodies with PRNT assay is generally accepted as a surrogate measure for efficacious JEV immunity [9]. Despite the widespread use of JE vaccines and the circulation of heterologous genotypes, vaccine-induced neutralization capacity has mostly been assessed solely against the strain homologous to the vaccine strain [10–12]. It is well known, however, that the various strains and genotypes exhibit antigenic differences [13]. Cross-protection elicited by JE vaccines has mainly been addressed by JEV challenge studies in animals; these suggest variable levels of protection against challenge with heterologous JEV [14–16]. In humans, differences have been found in the vaccine-induced immune response to heterologous virus strains even within the same genotype (GIII) after primary immunization with the live-attenuated [17], and the inactivated mouse brain [8, 13, 17] or Vero cell–derived vaccines [8].

Human data on immune response against strains of heterologous genotypes are scarce [18, 19]. To our knowledge, this is the first study to explore the cross-reactive potential of the inactivated JE vaccines against nonvaccine genotypes in travelers, and the first human study to address the cross-protection elicited by the new inactivated SA14-14-2–based vaccine, Ixiaro.

Our study shows protective levels of cross-reactive neutralizing antibodies to genotypes I–IV in European travelers after a primary series with inactivated SA14-14-2–based and Nakayama-based vaccines, implying good cross-protective capacity for both of these preparations against all major genotypes currently circulating. The GV strain was not available for testing, yet, as long as GV remains such a rare cause of encephalitis, this genotype appears to be of minor clinical significance.

Our study also showed differences in the levels of neutralizing antibodies against various genotypes: interestingly, the most pronounced immune response was observed against a strain representing GII, which is heterologous to both vaccine strains. The second-highest titers were seen against the strains homologous to those in the vaccines, whereas responses to GI and GIV remained somewhat lower. Notably, the low cross-protection found against GI, the genotype emerging as the most prevalent genotype in many areas of Asia [3, 5, 6], calls for special attention in the future. Importantly, despite the differences between the genotypes, responses to all genotypes reached protective levels.

The majority of all JE cases are encountered in areas where JE vaccines have been implemented in the national vaccination program [1]. A marked decrease has been seen in the JE incidence after the introduction of childhood vaccinations [1], which supports our findings on the cross-protective capacity of GIII-derived vaccines.

The present study shows that the inactivated JE vaccines currently given to travelers provide significant protection against the most important JEV genotypes circulating in endemic countries at the moment; however, no data are available on the duration of this cross-protection. This should be addressed in future studies in nonendemic populations where natural boosters can be excluded. Special attention should be paid to the longevity of the cross-protective response to GI.

The present study is the first human study to explore the immunogenicity of the new JE vaccine Ixiaro against heterologous genotypes, and the first to explore the cross-genotype immunogenicity of the inactivated JE vaccines in travelers. Our data show a significant cross-protective capacity against heterologous strains representing genotypes I–IV. This implies that, at present, both the inactivated JE vaccines given to travelers can be expected to confer protection against all major genotypes found in endemic areas.
